# Using Dried Blood Spots to Quantitatively Detect Anti–SARS-CoV-2 IgG Antibodies by ELISA: A Validation Study

**DOI:** 10.4269/ajtmh.23-0306

**Published:** 2024-06-25

**Authors:** Michelle Ylade, Asma Binte Aziz, Jedas Veronica Daag, Maria Vinna Crisostomo, Kristal-An Agrupis, Maria Angela Maronilla, Chloe Sye Lim Hong, Hwa Young Kim, Irene Njau, March Helena Jane Lopez, Jacqueline Deen, Deok Ryun Kim, Young Ae You, Sophie S. Y. Kang, Florian Marks, Birkneh Tilahun Tadesse

**Affiliations:** ^1^ Institute of Child Health and Human Development, National Institutes of Health, University of the Philippines–Manila, Manila, Philippines;; ^2^ International Vaccine Institute, Seoul, Korea;; ^3^ Cambridge Institute of Therapeutic Immunology and Infectious Disease, University of Cambridge School of Clinical Medicine, Cambridge Biomedical Campus, Cambridge, United Kingdom;; ^4^ Heidelberg Institute of Global Health, University of Heidelberg, Heidelberg, Germany;; ^5^ Madagascar Institute for Vaccine Research, University of Antananarivo, Antananarivo, Madagascar;; ^6^ Department of Global Public Health, Karolinska Institutet, Stockholm, Sweden

## Abstract

SARS-CoV-2 serological testing is useful to determine seroprevalence, epidemiological trends, and the extent of transmission. The collection and transport of serum samples can be logistically challenging, especially in remote underserved areas. Dried blood spots (DBSs) would allow easier sample collection and logistical handling compared with standard serum collection, particularly for extensive and repeated SARS-CoV-2 serosurveys. We evaluated the sensitivity, specificity, positive predictive value (PPV), and negative predictive value (NPV) of the IgG ELISA (Wantai, Beijing, China) using DBSs against sera for the quantitative detection of SARS-CoV-2 IgG antibody. The IgG ELISA was used to test paired sera and DBSs obtained from individuals with recent virologically confirmed COVID-19 illness and banked paired sera and DBSs collected before the COVID-19 pandemic. We found that 100/100 (100%) seropositive samples were positive using DBSs, and 193/194 (99%) seronegative samples were negative using DBSs. Compared with sera, the DBS method had a 100% sensitivity, 99% specificity, 99% PPV, and 100% NPV. Use of DBSs for SARS-CoV-2 household or population serosurveys may be considered in situations with limitations in sample collection, shipment, and storage.

## INTRODUCTION

Aside from the detection and reporting of symptomatic COVID-19 cases, serological screening is an important epidemiological tool for monitoring SARS-CoV-2 infections in the individual and community. SARS-CoV-2 household or population surveys may be used to assess seroprevalence or gain insight into the spread of the virus over time and across age groups and geographic regions.^1^ The ELISA, with a turnaround time of 2–8 hours, is the most commonly used serological test.^2^ The Wantai SARS-CoV-2 IgG ELISA (Beijing, China) was developed for the quantitative detection of IgG antibodies to SARS-CoV-2 in sera or plasma after immunization or prior infection. A recent evaluation of 11 SARS-CoV-2 antibody tests was conducted using a reference set of serum and plasma samples from unexposed persons and COVID-19 patients.^3^ The Wantai SARS-CoV-2 antibody ELISA was found to have a specificity of 100% (95% CI: 98–100%) and overall sensitivity of 89% (95% CI: 83–93%).^3^

Dried blood spots (DBSs) provide a reliable and simple alternative to serum or plasma for conducting serological testing. It can potentially be used to assess neutralizing antibody responses as a key correlate of protection against SARS-CoV-2.^4^ Using DBSs for ELISA instead of sera could make large and repeated SARS-CoV-2 serosurveys, especially in rural areas and low-resource settings, more accessible for sample collection, shipment, transport, and storage. Although the comparison of DBSs against sera for detecting SARS-CoV-2–specific IgG by various ELISAs has been previously reported,^5^^,^^6^ the performance of DBSs using the commercially available Wantai SARS-CoV-2 antibody ELISA kit has not been investigated.

## MATERIALS AND METHODS

This diagnostic accuracy study compared the quantitative detection of SARS-CoV-2–specific IgG by Wantai ELISA using DBSs versus sera. The study followed the Standards for the Reporting of Diagnostic Accuracy Studies guidelines. The protocol was reviewed and approved by the University of the Philippines–Manila Research Ethics Board and the institutional review board of the International Vaccine Institute. The participants provided written informed consent. The study was conducted in accordance with the principles of the Declaration of Helsinki.

### Study participants and sample collection.

We tested paired DBSs and sera from SARS-CoV-2–seropositive and –seronegative individuals. The seropositive reference set of paired sera and DBS samples was obtained in March 2022 from adult participants in Metro Manila, Philippines, with a history of SARS-CoV-2 infection (diagnosed by reverse transcription polymerase chain reaction within 3 months from the date of enrollment), irrespective of COVID-19 vaccination status. For the seronegative reference set, we used archived paired sera and DBS samples collected in May–June 2017 from children 9–14 years of age in Cebu, Philippines (prior to the start of the COVID-19 pandemic) as part of an ongoing dengue cohort study (ClinicalTrials.gov, #NCT03465254).^7^ The participants provided written informed consent for the storage and future use of data and specimens.

From participants in both the seropositive and seronegative reference sets, 5 mL of blood was collected in anticoagulant-free vacutainer tubes, of which 60–70 *µ*L of blood was blotted at the center of each collection card (Whatman 903 filter paper; Global Life Sciences Solutions USA LLC, Wilmington, DE) using a disposable transfer pipette. The remaining blood was processed, and the serum was aliquoted. The blood on the filter paper was allowed to air-dry for at least 4 hours on a rack. After completion of the drying process, the DBSs were packed individually in single, gas-permeable zipper bags with three to five desiccant sachets and a humidity indicator. The sera and DBSs were stored at −80°C before testing.

### Measurement of SARS-CoV-2 IgG antibodies.

The sera and DBSs in both the seropositive and seronegative reference sets were batch tested from May 2022 to June 2022. A trained medical technologist performed the laboratory procedures. The prospectively collected and archived samples were tested using the Wantai SARS-CoV-2 IgG ELISA. For the assay, 10 *µ*L of serum was used. Dried blood spots were eluted prior to IgG ELISA testing. One circle of dried blood on filter paper was punched into a microcentrifuge tube using a 6-mm device puncher (Whatman WB100040 Harris Uni-Core Punch; Global Life Sciences Solutions USA LLC). A single spot of dried blood yields approximately three 6-mm filter paper circles. The number of sera in each filter paper circle was estimated using the following formula: 0.5 × (the volume of blood per spot divided by the number of filter paper circles per spot). Thus, each filter paper circle contained approximately 10–12 *µ*L of sera (0.5 × (60–70 *µ*L/3)), which is comparable to the required amount (10 *µ*L) in serum ELISA testing.

In the Wantai kit, polystyrene microwell strips are precoated with SARS-CoV-2 recombinant antigen. During the first incubation step, SARS-CoV-2 IgG antibodies, if present, were bound to the solid-phase precoated antigens. The wells were washed to remove unbound serum proteins; then, anti-human IgG antibodies (anti-IgG) conjugated to horseradish peroxidase (HRP-conjugate) were added. During the second incubation step, these HRP-conjugated antibodies were bound to antigen-antibody (IgG) complexes previously formed, and the unbound HRP-conjugate was then removed by washing. Chromogen solutions containing tetramethyl benzidine and urea peroxide were added to the wells. In the presence of the antigen-antibody–anti-IgG (HRP) immunocomplex, the colorless chromogens were hydrolyzed by the bound HRP-conjugate to a blue-colored product. The blue color turned yellow after the reaction was stopped with sulfuric acid. Wells containing specimens negative for SARS-CoV-2 IgG remained colorless. The ELISA optical density (OD) was used to compute the IgG level as follows. The logarithmic value (ln) of the OD of the specimen was substituted into a linear regression equation to get the antibody concentration of the corresponding sample. A linear regression equation was obtained using the standard antibody concentration and the mean value of its corresponding absorbance values to do a double logarithmic curve. An IgG level of <1.0 U/mL was interpreted as negative and ≥1.0 U/mL as positive (indicating the presence of detectable IgG antibodies from a past or recent SARS-CoV-2 infection or vaccination). Because the Wantai ELISA is for the quantitative detection of SARS-CoV-2 IgG (positive or negative), when the absorbance value of the sample exceeded the linear portion of the standard curve of the serially diluted comparator from which the ELISA IgG levels are determined (overflow), no dilution and retesting of the sample was done. The overflow samples were considered as having a SARS-CoV-2 IgG level of >32.0 U/mL and were reported as positive.

## STATISTICAL ANALYSES

For the validation of the ELISA using DBSs compared with sera, an assumed lower sensitivity and specificity of the ELISA using DBSs of 10% was considered. We determined the minimum sample size required to provide 80% power at a 95% CI for the evaluation of both sensitivity and specificity^8^ as 100 seropositive samples and 200 seronegative samples, including an additional 3% for inadequate or inappropriate samples.

We compared the demographic characteristics of SARS-CoV-2–seropositive and –seronegative participants using a *t-*test for continuous variables and χ^2^ test for categorical variables. We described the clinical features and COVID-19 vaccination history of participants with recent COVID-19 illness. We calculated the sensitivity, specificity, positive predictive value (PPV), negative predictive value (NPV), and number of false-negatives and false-positives of the SARS-CoV-2 IgG ELISA results using DBSs compared with that using sera. To evaluate the correlation between DBSs and sera ELISA results, we carried out the Cohen’s κ statistics for categorical values and Lin’s concordance correlation coefficient for continuous values. We used the Clopper-Pearson exact method for calculating 95% CIs for the performance indicators on categorical data. We also did a receiver operating characteristics (ROC) analysis and calculated the area under the curve (AUC), which was considered an aggregate measure of the accuracy of the ELISA using DBSs compared with sera. An AUC of 1.0 is ideal while an AUV greater than 0.8 is considered acceptable.^9^ We used the Wilcoxon signed-rank test to compare mean IgG levels from DBSs and serum samples. We used Stata 17.0 (StataCorp, 2015, College Station, TX) to compare the participant groups. We used the epiR library of the R package to calculate the performance indicators.^10^

## RESULTS

**Figure 1.**
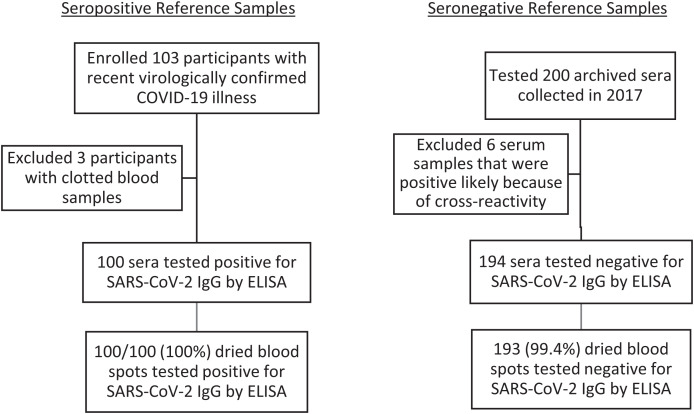
Schema of specimens collected.

We enrolled, collected a blood sample from, and tested the sera from 103 participants with a recent history of SARS-CoV-2 infection (excluding three with clotted samples) and tested randomly selected archived sera from 200 participants (Figure 1). All 100 sera samples from participants with recent COVID-19 were positive for SARS-CoV-2 IgG by ELISA, of whom 88/100 (88%) had SARS-CoV-2 IgG levels of >32 ELISA units. Of the archived sera collected before the COVID-19 pandemic, we excluded six with positive results, which were likely due to cross-reactivity with non–SARS-COV-2 coronaviruses, and included 194 with seronegative results.

We compared the demographic characteristics of the 100 and 194 participants whose sera samples were positive and negative for SARS-CoV-2 IgG by ELISA, respectively (Table 1). The seropositive and seronegative reference sets comprised adults and older children, respectively.

**Table 1:** Comparison* of demographic characteristics of all participants whose serum and dried blood spot samples were tested by SARS-CoV-2 IgG ELISA

Demographic Characteristics	Participants Whose Sera Were Positive for SARS-CoV-2 IgG by ELISA (*N* = 100)	Participants Whose Sera Were Negative for SARS-CoV-2 IgG by ELISA (*N* = 194)	*P*-Value
Sex			
Male, *n* (%)	31 (31.0)	93 (47.9)	0.005
Female, *n* (%)	69 (69.0)	101 (52.1)	
Age (in years)			
Mean (SD)	36.1 (11.20)	10.8 (1.51)	<0.0001
Median (IQR)	33 (18)	11 (3)	

IQR = interquartile range.

* We used the *t-*test for continuous variables (age) and χ^2^ test for categorical variables (sex).

The clinical features of the 100 participants in the seropositive reference set are shown in Table 2. The majority, 96/100 (96%), had their COVID-19 illness within 2 months of sample collection. The most common manifestations were sore throat (68%), cough (66%), and fever (47%). One participant had been admitted to the hospital for the illness. Of the 100 participants in the seropositive reference set, 98 (98%) had received two doses of COVID-19 vaccine, with the Oxford/Astra Zeneca COVID-19 vaccine being the most common (Table 3). Sixty-nine of 98 (70%) and 70 of 98 (71%) participants received their first and second COVID-19 doses, respectively, at a hospital vaccination site, whereas the remainder were vaccinated at a local government unit vaccination site, with very few at a clinical trial facility (Table 3).

**Table 2:** Clinical features of participants with recent COVID-19 illness (*N* = 100)

Clinical Features	Frequency (%)
Date of COVID-19 illness	
December 2021	4/100 (4)
January 2022	96/100 (96)
Presence of any symptoms	95/100 (95)
Signs and symptoms	
Sore throat	68 (68)
Cough	66 (66)
Fever	47 (47)
Headache	50 (50)
Fatigue	44 (44)
Muscle pain	40 (40)
Diarrhea	17 (17)
Loss of taste	17 (17)
Loss of smell	15 (15)
Difficulty breathing	10 (10)
Vomiting	5 (5)
Asymptomatic	5 (5)
Admitted to hospital	1 (1)

**Table 3:** COVID-19 vaccination details of participants with recent COVID-19 illness

COVID-19 Vaccination	Frequency (%)
Receipt of COVID-19 vaccine	
First dose	98/100 (98)
Second dose	98/100 (98)
Third dose	74/100 (74)
COVID-19 vaccine brand	
Oxford/Astra Zeneca (ChAdOx1-S [recombinant vaccine])	
First dose	42/98 (43)
Second dose	41/98 (42)
Third dose	5/74 (7)
Moderna mRNA	
First dose	16/98 (16)
Second dose	16/98 (16)
Third dose	17/74 (23)
SCB-2019/CLOVER	
First dose	2/98 (2)
Second dose	2/98 (2)
Third dose	1/74 (1)
Sinovac-CoronaVac	
First dose	38/98 (39)
Second dose	39/98 (40)
Third dose	0/74 (0)
Pfizer-BioNTech	
First dose	0/98 (0)
Second dose	0/98 (0)
Third dose	51/74 (69)
COVID-19 vaccination facility	
Local government unit vaccination site	
First dose	27/98 (28)
Second dose	26/98 (27)
Third dose	16/74 (22)
Hospital vaccination site	
First dose	69/98 (70)
Second dose	70/98 (71)
Third dose	57/74 (77)
Clinical trial facility	
First dose	2/98 (2)
Second dose	2/98 (2)
Third dose	1/74 (1)

mRNA = messenger RNA.

**Figure 2.**
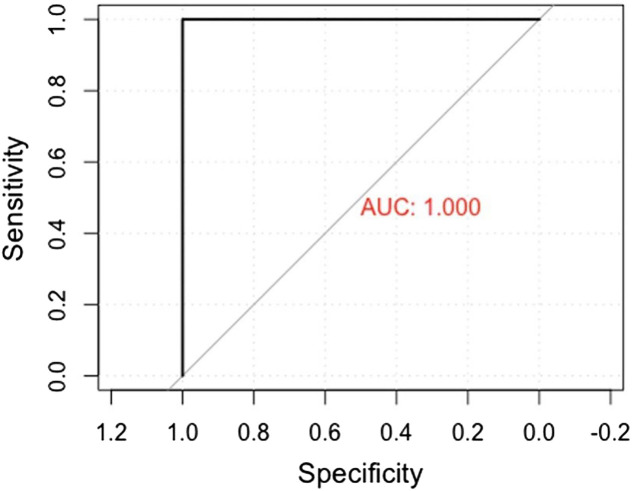
Receiver operating characteristics curve and area under the curve (AUC).

**Figure 3.**
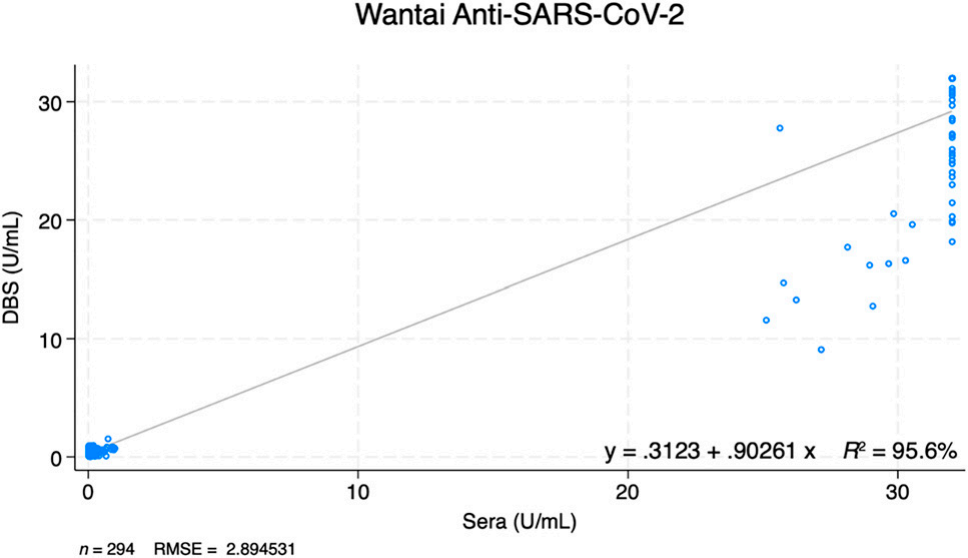
Correlation between anti–SARS-CoV-2 IgG level using dried blood spots (DBSs) and sera. RMSE = root mean square error.

One hundred of 100 (100%) of the seropositive sera samples were also positive using DBSs and 193/194 (99%) seronegative sera samples were negative using DBSs (Figure 1; Table 4). The DBS method had a 100% (96–100%) sensitivity, 99% (97–100%) specificity, 99% (93–100%) PPV, and 100% (98–100%) NPV compared with sera (Table 4). The Cohen’s κ value was 99.2% (97.8–100%). The ROC curve showed an AUC of 1.0 (Figure 2). SARS-CoV-2 IgG levels from DBSs were compared with those of serum samples (Figure 3). There was high correlation between quantitative results obtained on DBS and sera. Lin’s concordance correlation coefficient was 97.3% (96.7–97.8%).

**Table 4:** Performance of SARS-CoV-2 IgG ELISA using dried blood spots compared with serum samples

		Serum Samples, *n* (%)		Total
		Seropositive	Seronegative	
Dried blood spots	Seropositive	100 (100)	1 (0.5)	101 (34.4)
	Seronegative	0 (0)	193 (99.5)	193 (65.6)
	Total	100 (100)	194 (100)	294 (100)
Sensitivity (95% CI): 100.0% (96.4–100.0%) Specificity (95% CI): 99.5% (97.2–100.0%) Positive Predicted Value (95% CI): 99.0% (93.4–99.9%) Negative Predicted Value (95% CI): 100.0% (97.6–100.0%) False-Negative (95% CI): 0% (0–3.6%) False-Positive (95% CI): 0.5% (0–2.8%) Cohen’s κ Value (95% CI): 99.2% (97.8–100%) Lin’s Concordance Correlation Coefficient (95% CI): 97.3% (96.7–97.8%)				

## DISCUSSION

We found that the IgG ELISA qualitative and quantitative results using DBSs were very closely correlated with the results when using sera, with a slight reduction in specificity. The DBS results were highly accurate, as indicated by the AUC of 1.0. Our findings are similar to those obtained from previous studies.^5^^,^^6^ Matched serum and DBS samples from 52 healthcare workers were tested using a commercially available ELISA (Euroimmun Medizinische Labordiagnostika, Lubeck, Germany) for the presence of anti–SARS-CoV-2 IgG and the qualitative results showed good concordance.^6^ In another study, there was a significant correlation of SARS-CoV-2 IgG, IgM, and IgA levels between matched serum and DBS samples from 80 volunteers tested using an in-house ELISA, with 98% sensitivity and 100% specificity.^5^ Another study compared paired serum and DBS samples from adult volunteers who were either SARS-CoV-2 RT-PCR positive or negative at least 2 weeks prior to blood collection. This study suggested the need for confirmatory testing on positive results.^11^ Still, DBS ELISA has been used in SARS-CoV-2 household transmission studies^12^ and for monitoring of SARS-CoV-2 antibodies^13^ and has been assessed for suitability in large population serosurveys.^14^

Dried blood spot sampling is easy to perform in the field and requires minimal equipment and training. We stored our DBSs at −80°C, but others found that DBS specimens were stable for at least 28 days at ambient room temperature and humidity.^15^ One limitation of our study was that the DBSs were from venous blood blotted onto filter paper rather than from finger prick, so our results may not reflect the performance when DBSs are collected using capillary blood samples. Dried blood spots from capillary blood samples are likely to generate variable test results because of smaller volumes of blood collected via DBS. This may result in false-negative results.^16^ The participants in the seropositive and seronegative reference sets belonged to different age groups; however, this is unlikely to affect the overall results. The majority of our seropositive participants had SARS-CoV-2 IgG levels of >32, and our results may not be generalizable to other populations with lower IgG levels.

## CONCLUSION

In summary, we found that the quantitative detection of SARS-CoV-2 IgG by Wantai ELISA was comparable when using DBSs versus sera. Dried blood spots may be a useful alternative to sera for SARS-CoV-2 household or population serosurveys.
